# Self‐Management Improves Long‐Term CKD Prognosis: A 10‐Year Retrospective Cohort Study From China

**DOI:** 10.1155/jonm/1228799

**Published:** 2026-03-03

**Authors:** Hui-fen Chen, Xing-le Liang, Yan Han, Yu-han Shen, Xian-long Zhang, Fang Tang, Li-zhe Fu, Yue-yu Gu, Tao Zou, Xin-dong Qin, Wen-wei OuYang, Xu-sheng Liu, Yi-fan Wu

**Affiliations:** ^1^ Chinese Medicine Guangdong Laboratory, Department of Nephrology, The Second Clinical College, Guangzhou University of Chinese Medicine (Guangdong Provincial Hospital of Chinese Medicine), Guangzhou, Guangdong, China; ^2^ School of Nursing, Guangzhou University of Chinese Medicine, Guangzhou, Guangdong, China, gzucm.edu.cn; ^3^ Department of Chronic Disease Management, The Second Clinical College, Guangzhou University of Chinese Medicine (Guangdong Provincial Hospital of Chinese Medicine), Guangzhou, Guangdong, China; ^4^ State Key Laboratory of Traditional Chinese Medicine Syndrome, The Second Clinical College, Guangzhou University of Chinese Medicine (Guangdong Provincial Hospital of Chinese Medicine), Guangzhou, Guangdong, China; ^5^ Key Unit of Methodology in Clinical Research, The Second Clinical College, Guangzhou University of Chinese Medicine (Guangdong Provincial Hospital of Chinese Medicine), Guangzhou, China; ^6^ Global Health—Health Systems and Policy, Department of Global Public Health, Karolinska Institute, Stockholm, Sweden, ki.se

**Keywords:** chronic kidney disease, disease progression, longitudinal data, self-management

## Abstract

**Background:**

Self‐management (SMP) is a novel treatment comprised of multidimensional interventions. Researchers have reported its reliable effects on short‐term improvements in surrogate indicators like 24‐h urinary protein excretion and systolic blood pressure. However, whether it has any effect on long‐term changes in other surrogate indicators and chronic kidney disease (CKD) endpoints remains unclear.

**Methods:**

Patients with CKD stages 3‐4 were grouped into either a SMP group or a nonself‐management (non‐SMP) group whether they had complied any of five characterized elements: regular visits, medication adjustment, nutritional intervention, lifestyle modification, education, and behavior guide. We used 1:1 nearest‐neighbor propensity score matching (PSM) to balance between‐group differences. Cox regression was used to explore the long‐term association between SMP and composite outcomes (all‐cause mortality, end‐stage kidney disease (ESKD), ≥ 50% decline in estimated glomerular filtration rate (eGFR), or doubling of serum creatinine (SCr) from baseline). Linear mixed‐effects (LME) model and marginal means comparisons were used to surrogate indicators analysis.

**Results:**

The study included 1160 patients with a median follow‐up of 27.20 months. Among them, 580 individuals were in the SMP group and 580 were in the non‐SMP group. In the non‐SMP group, the median follow‐up was 24.48 (12.21, 43.39) months with 174 (30.00%) events, whereas the SMP group had a median follow‐up of 29.34 (14.75, 42.00) months with 134 (23.10%) composite outcomes. Participation in a SMP program was associated with a 30.9% reduction in CKD progression risk (hazard ratio (HR) of composite outcome: 0.691, 95% CI: 0.549–0.869, and *p* = 0.002). LME and marginal means comparison indicated that patients with SMP had higher mean difference of eGFR and serum albumin (Alb) than the non‐SMP individuals (mean difference of eGFR: 1.791 (0.721, 2.861) mL/min/1.73 m^2^, *p* = 0.001; marginal mean difference of eGFR: 2.560 (1.660, 3.470) mL/min/1.73 m^2^, *p* < 0.0001; mean difference of Alb: 0.654 (0.202, 1.105) g/L, *p* = 0.005; and marginal mean difference of Alb: 0.460 (0.166, 0.753) g/L, *p* = 0.002).

**Conclusion:**

SMP programs are associated with a 30.9% reduction in CKD progression risk and improvements in kidney function and nutrition‐related surrogate markers, benefiting CKD patients in stages 3‐4.

## 1. Introduction

Chronic kidney disease (CKD) is a noncommunicable disease affecting 10%–35% of adults globally [1]. It has a prevalence rate of 8.2% in Mainland China [2]. Multiple organs and systems become involved as the disease progresses. Once it progresses to end‐stage kidney disease (ESKD), renal replacement therapy (RRT) (dialysis and transplantation) is necessary for patients with CKD to prolong their lives. Thus, exploring effective and reliable treatments for thwarting CKD progression is crucial to current clinical study.

Traditional treatment methods used in clinical practice for CKD include medications such as hormones and immunosuppressants, as well as angiotensin‐converting enzyme inhibitors (ACEi)/angiotensin II receptor blockers (ARBs) [3, 4]. The application of new drugs, such as sodium–glucose cotransporter 2 inhibitors (SGLT2i), canakinumab, and mineralocorticoid antagonists (Finerenone) also plays an important role in preventing CKD progression [5–7]. However, CKD is closely associated with lifestyle. Without lifestyle adjustments, the efficacy of medication‐centered treatments may plummet [8, 9]. Numerous studies have indicated that nutrition management, exercise, improvement in sleep, and even improvement in medication adherence have positive effects on CKD [5, 10–12]. Correcting these unhealthy lifestyle and medication habits requires improving CKD patients’ self‐management (SMP) skills.

SMP is the voluntary practice of activities initiated and performed by individuals to maintain their life, health, and well‐being [13]. For patients with chronic diseases, individuals need to engage in a series of activities, including health goal setting, self‐monitoring, decision‐making, problem solving, behavioral planning and implementation, and self‐assessment, in order to possess the capacity to regulate the physical, emotional, and cognitive responses associated with the disease, which enables them to exert comprehensive control over chronic diseases and related risk factors [14–16]. Studies have indicated that SMP can mitigate risk factors, alleviate kidney disease symptoms, improve motor function, enhance self‐efficacy, and improve anxiety [17–19]. Devins et al. also found that compared with usual care, a predialysis educational intervention could slow disease progression and delay the need for RRT by a median of 17.0 months in non‐dialysis CKD patients [20, 21]. SMP gradually sprang up in the field of CKD in China in the late 2010s, leading to a boom in related research [22, 23]. However, our team’s 2019 meta‐analysis showed that related studies had a mean follow‐up duration of 13.44 months, which may be too short to assess the real effects of CKD SMP. Additionally, we only detected effects on surrogate indicators like 24‐h urinary protein excretion and systolic blood pressure [24]. This raises several questions: Did the long‐term continuous SMP lead to more improvement in other surrogate indicators such as hemoglobin (Hb) and albumin (Alb)? Could it further improve the endpoints of CKD prognosis?

Our team has been implementing systematic SMP for CKD for a long time and has retained a large amount of real‐world data on CKD clinical diagnosis and treatment (Supporting file 1). In this study, we have collected longitudinal data on CKD adults to explore the long‐term efficacy of SMP on CKD surrogate indicators and prognosis.

## 2. Methods

### 2.1. Study Design

In this retrospective cohort study, we searched the Hospital Information System (HIS) at Guangdong Provincial Hospital of Chinese Medicine (GPHCM) with the keywords “kidney disease,” “renal failure,” “renal diseases,” “nephritis,” “proteinuria,” and “hematuria.” Patients with kidney disease who visited from March 2012 to March 2023 were enrolled. This study protocol was reviewed and approved by the Ethics Committee at Guangdong Provincial Hospital of Chinese Medicine (no. ZE2023‐330).

We included patients aged 18–80 years, with CKD stages 3‐4. We excluded any patients diagnosed with acute or critical illnesses (e.g., acute cerebral infarction, acute heart failure, shock, malignant tumors, or hematological diseases), who had received RRT within 3 months from the baseline, who had estimated glomerular filtration rate (eGFR) less than 5 mL/min/1.73 m^2^, who had a follow‐up period less than 3 months, or who had incomplete follow‐up data. CKD stages were calculated according to the CKD Epidemiology Collaboration (CKD‐EPI) creatinine equation [25].

### 2.2. Screening Process

**FIGURE 1:**
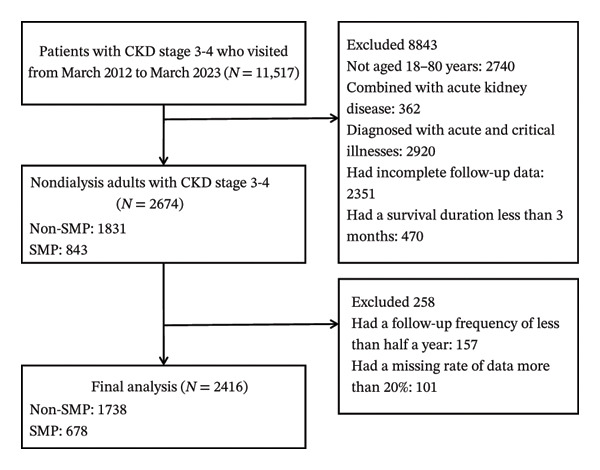
Patient screening flowchart showing recruitment, exclusions, and final analytic sample (*n* = 2416).

A total of 11,517 individuals with CKD stages 3‐4 visited GPHCM between March 2012 and March 2023. A total of 2740 individuals were not aged 18–80 years old. A total of 362 were comorbid with acute kidney disease. A total of 2920 had been diagnosed with acute and/or critical illnesses. A total of 2351 had incomplete follow‐up data and 470 had a survival duration less than 3 months. At first, 2674 adults with CKD stages 3‐4 were enrolled. A total of 1831 were in the non‐SMP group and 843 were in the SMP group. A total of 157 individuals had a follow‐up frequency of less than half a year. A total of 101 had a missing data rate over 20%. A total of 2416 individuals in total were included, with 678 SMP and 1738 non‐SMP cases (Figure 1).

### 2.3. Defining the Exposures

SMP, a specialty service at GPHCM launched in 2009 and standardized from 2012, consisted of five elements: regular visits, medication adjustment, nutritional intervention, lifestyle modification, education, and behavior guide. Participants were required to visit every three to six months based on their conditions. Patients were grouped into either a SMP group or a non‐SMP group according to whether they had complied any of above five elements. Patients with a follow‐up frequency of less than half a year were excluded to ensure that the patients included in the final analysis had good follow‐up compliance. Introduction of our SMP service is provided in Supporting file 1 and Reference [14].

### 2.4. Data Collection

Covariates included demographic characteristics, clinical diagnosis information, and laboratory measurements (Supporting file 2). Covariates with a missing rate of 40% or more at baseline (phosphorous (P), calcium (Ca^2+^), potassium (K^+^), sodium (Na^+^), fasting blood glucose (FBG), and urine protein/creatinine ratio (PCR)) were excluded from the final analysis. Covariates were collected every 6 months until the occurrence of an adverse event of interest or dropout from the study, whichever came first.

### 2.5. Definition of Endpoints

Composite outcome was defined as all‐cause mortality, ESKD (received RRT or eGFR < 15 mL/min/1.73 m^2^), ≥ 50% decline in eGFR, or doubling of SCr from baseline. Patients were followed to the end of the follow‐up time period (March, 2023) or loss to follow‐up (defined as being unobservable during the time‐at‐risk period).

### 2.6. Statistical Analyses

Outliers of continuous variables were winsorized with 0.1 cutoffs at each tail. Missing baseline covariates were imputed with multiple imputation for the whole dataset and pooled the estimators across imputed datasets (with the MatchThem R package). For repeated measurements, missing data were imputed using the mean value. Propensity scores were calculated as the probability of undergoing SMP compared with non‐SMP using the logit method and adjusting for any relevant confounders. Propensity score matching (PSM) was performed at a 1:1 ratio by a nearest‐matching technique to balance any between‐group difference (caliper was 0.20). Continuous variables were described using the mean ± standard deviation (SD) for normal distributions and median (interquartile ranges) for nonnormal distributions. Categorical variables were reported by proportions. A Mann–Whitney *U*‐test and a chi‐square test were applied to compare characteristics between groups.

Survival probability was compared using the Kaplan–Meier curve and a log‐rank test. Univariate Cox regression was used for preliminary screening of potential variables associated with composite outcome. Multivariate Cox models were fitted with to explore the association between exposures at baseline and composite outcome. Model 1 was based on univariate Cox regression of SMP vs. non‐SMP. Model 2 further adjusted selected features in least absolute shrinkage and selection operator (LASSO) regression combined with cross‐validation. Subgroup analysis with interaction effects were constructed by stratification of age, sex, PCR, CKD stage, and hypertension and diabetes status. The results were presented in a forest plot.

In order to further explore potential mechanisms of SMP, we constructed linear mixed‐effects (LME) model and marginal means comparison to better understand the difference between the significant surrogates eGFR, BUN, Hb, Alb, and TC between the SMP and non‐SMP groups (with the lme4 R package).

Proportional hazard (PH) assumption was tested via Schoenfeld residuals. An interaction with time of follow‐up was introduced if the PH assumption was violated. Associations were assessed using hazard ratios (HRs) and a 95% confidence interval (95% CI). For variable selection in multivariate Cox regression, variables with *p* < 0.10 in univariate analysis were included. All other statistical tests were two‐sided, and *p* values < 0.05 were considered statistically significant. Statistical analysis was performed with R Version 4.2.1.

## 3. Results

### 3.1. Basic Clinical Features

After adjusting all candidate variables using PSM, a total of 1160 individuals with a median age of 59.69 (48.64, 68.58) years, and 543 (46.81%) females were included in the final analysis. A total of 465 (40.09%), 345 (29.74%), and 350 (30.17%) were with CKD stages 3a, 3b, and 4, respectively. A total of 348 (30.00) had been diagnosed with primary glomerulonephritis. A total of 34 (2.93%) had hypertensive renal disease and 66 (5.69%) had diabetic nephropathy. A total of 93 (8.02%) had other secondary kidney diseases and 619 (53.36%) were estimated to have had unknown etiology. A total of 879 (75.78%) patients had a history of hypertension, 529 (45.60%) had a history of diabetes, 454 (39.14%) had been diagnosed with hyperlipidemia, and 579 (49.91%) had been diagnosed with hyperuricemia. A total of 476 (41.03%) had been diagnosed with anemia. A total of 202 (17.41%) had been diagnosed with CVDs. A total of 441 (38.02%) of them reported the use of ACEI/ARB. The baseline characteristics are described in Supporting Table 1.

### 3.2. Survival Analysis

**FIGURE 2:**
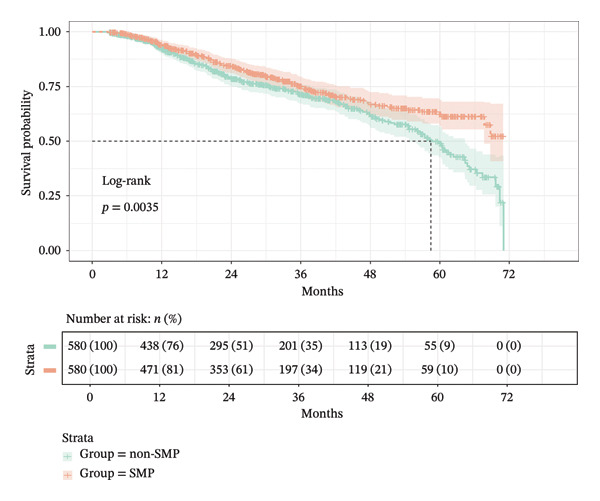
Kaplan–Meier survival curves comparing non‐SMP and SMP groups with log‐rank test (*p* = 0.0035), showing significantly different survival probabilities over 72 months.

The cohort had a median follow‐up duration of 27.20 (13.15, 42.66) months, and 308 (26.55%) subjects reached the composite outcome, comprising all‐cause mortality (*n* = 8, 2.6%), ESKD (*n* = 282, 91.56%), ≥ 50% decline in eGFR (*n* = 11, 3.6%), and doubling of SCr from baseline (*n* = 7, 2.3%). The median follow‐up period for the non‐SMP group was 24.48 (12.21, 43.39) months with 174 (30.00%) events of interest. SMP had a median follow‐up period of 29.34 (14.75, 42.00) months, with 134 (23.10%) composite outcomes. The median time to composite outcome for the non‐SMP group was 58.4 (54.7–64.4) months. The Kaplan–Meier curves showed that the SMP group had a more desirable survival probability than the non‐SMP group with a gradually increasing difference (HR = 0.716, 95% CI: 0.571–0.897, and *p* = 0.0035) (Figure 2).

### 3.3. Cox Model

Group, age, sex, eGFR, Hb, Alb, UA, BUN, TCO_2_, LDL‐C, TC, CKD stage, etiology, with hypertension, with diabetes, with anemia, with CVDs, use of sodium bicarbonate, diuretics and turbidity‐removing Chinese patent medicine were entered into the multivariate model (*p* < 0.10); BUN violated the PH assumption. Interactions with time of follow‐up were introduced for BUN (Supporting table 2). Using LASSO regression with cross‐validation and selecting the penalty at lambda.1se, the retained predictors with nonzero coefficients were group, age, sex, eGFR, Hb, Alb, BUN, TC, CKD stage, etiology, with hypertension, with diabetes, with CVDs, and use of diuretics; all other variables were shrunk to zero and excluded.

With non‐SMP as the control group, SMP had an HR of 0.716 (0.571, 0.897) (*p* = 0.004) in Model 1 and an HR of 0.691 (0.549, 0.869) (*p* = 0.002) after fully adjusting the covariates in Model 2 (Table 1). This indicated that SMP was a protective factor for preventing the occurrence of CKD composite endpoints. TC, with diabetes, and with CVDs were risk factors, and age, eGFR, Hb, and Alb were protective factors for CKD progression (*p* < 0.05).

**TABLE 1:** Multivariate cox regression.

**Variables**		**HR**	**p**

Model 1			
Group	Non‐SMP	Ref	
	SMP	0.716 (0.571, 0.897)	0.004

Model 2			
Group	Non‐SMP	Ref	
	SMP	0.691 (0.549, 0.869)	0.002

Sex	Males	Ref	
	Females	0.588 (0.460, 0.752)	< 0.001

Age, years		0.980 (0.972, 0.988)	< 0.001

eGFR, mL/min/1.73 m^2^		0.943 (0.932, 0.955)	< 0.001

Hb, g/L		0.988 (0.982, 0.994)	< 0.001

Alb, g/L		0.982 (0.965, 0.999)	0.039

BUN, mmol/L		1.026 (0.827, 1.272)	0.816

TC, mmol/L		1.116 (1.056, 1.180)	< 0.001

With hypertension	No	Ref	
	Yes	1.292 (0.935, 1.784)	0.120

With diabetes	No	Ref	
	Yes	1.800 (1.407, 2.303)	< 0.001

CVDs	No	Ref	
	Yes	1.603 (1.194, 2.152)	0.002

Diuretics	No	Ref	
	Yes	1.161 (0.844, 1.596)	0.359

*Note:* Model 1: univariate Cox regression of the group; Model 2: Model 1 + any significant variables from the LASSO regression, including group, age, sex, eGFR, Hb, Alb, BUN, TC, CKD stage, etiology, with hypertension, with diabetes, with CVDs, and use of diuretics.

### 3.4. Subgroup Analysis

**FIGURE 3:**
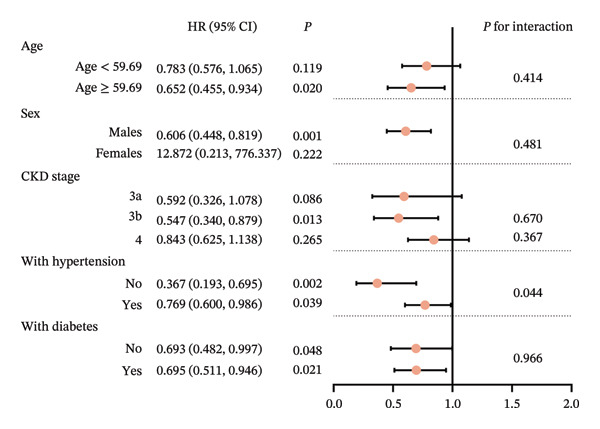
Forest plot showing HR with 95% confidence intervals for subgroup analysis of self‐management effects across age, sex, CKD stage, hypertension, and diabetes subgroups. Red dots represent HR, with horizontal lines indicating confidence intervals. Vertical line marks HR of 1.0.

Subgroup analysis showed that SMP had significant protective effects on the age ≥ 59.69 years subgroup (HR (95% CI):0.652 (0.455, 0.934), *p* = 0.020), males (HR (95% CI):0.606 (0.448, 0.819), *p* = 0.001), CKD stage 3b (HR (95% CI):0.547 (0.34, 0.879), *p* = 0.013), without hypertension (HR (95% CI): 0.367 (0.193, 0.695), *p* = 0.002), with hypertension (HR (95% CI): 0.769 (0.600, 0.986), *p* = 0.039), without diabetes (HR (95% CI): 0.693 (0.482, 0.997), *p* = 0.048), and with diabetes (HR (95% CI): 0.695 (0.511, 0.946), *p* = 0.021). Interaction exists between SMP and hypertension subgroups (*p* = 0.044) (Figure 3).

### 3.5. Potential Effects on the Surrogate Markers

Figure 4 visualized the changes in the mean values of eGFR, BUN, Hb, Alb, and TC during the follow‐up duration in both group. These surrogate markers showed nearly flat trends. Compared with the non‐SMP group, mean eGFR in the SMP was higher. Also, Hb’s mean was higher than those of the non‐SMP group for the same period (Figure 4).

FIGURE 4Longitudinal trajectories of five biological markers (eGFR, BUN, Hb, Alb, and TC) comparing non‐SMP and SMP groups across 14 visits (each visit represents 6 months), with estimated marginal means and error bars showing temporal changes in renal function and metabolic parameters over a 7‐year follow‐up period. Notes: left: illustrating the mean values derived from the original data; right: comparing the marginal means.

**Figure:**
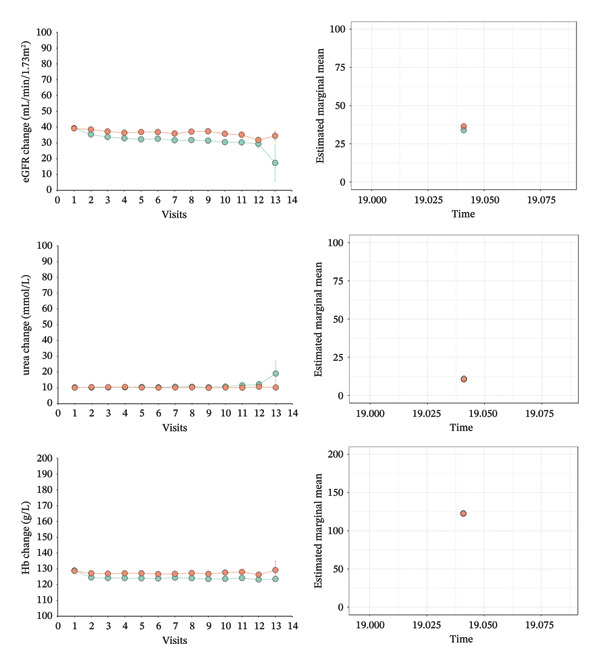
(a)

**Figure:**
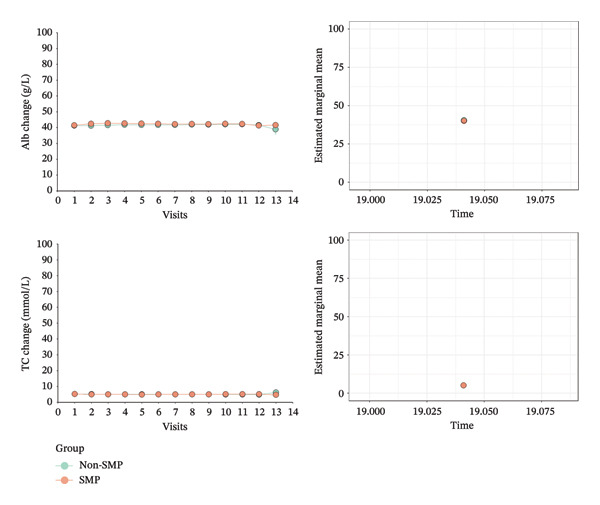
(b)

After adjusting significant variables in the previous multivariate Cox model, the LME models showed that the SMP group was 1.791 (0.721, 2.861) mL/min/1.73 m^2^ (*p* = 0.001) higher in eGFR mean difference, 0.405 (0.142, 0.668) mmol/L (*p* = 0.003) higher in BUN mean difference, and 0.654 (0.202, 1.105) g/L (*p* = 0.005) in Alb mean difference than the non‐SMP group during the follow‐up duration. The SMP group also was higher in Hb (mean difference: 0.219 (−1.262, 1.700) g/L) and lower in TC (mean difference: −0.009 (−0.125, 0.107) mmol/L), but this was not statistically significant (*p* value: Hb: 0.772 and TC: 0.881) (Table 2).

**TABLE 2:** Linear mixed‐effects model and marginal means comparison.

	**Linear mixed-effects model**			**Marginal means comparison**		
	**Mean difference (95% CI)**	***t***	**p**	**Mean difference (95% CI)**	***t***	**p**

eGFR, mL/min/1.73 m^2^	1.791 (0.721, 2.861)	3.281	0.001	2.560 (1.660, 3.470)	5.550	< 0.0001
BUN, mmol/L	0.405 (0.142, 0.668)	3.020	0.003	0.542 (0.321, 0.764)	4.803	< 0.0001
Hb, g/L	0.219 (−1.262, 1.700)	0.290	0.772	0.626 (−0.446, 1.700)	1.146	0.252
Alb, g/L	0.654 (0.202, 1.105)	2.835	0.005	0.460 (0.166, 0.753)	3.076	0.002
TC, mmol/L	−0.009 (−0.125, 0.107)	−0.150	0.881	−0.018 (−0.091, 0.055)	−0.474	0.636

After comprehensively considering the mean values of covariates and random effects, marginal means comparison also showed that the SMP group was 2.560 (1.660, 3.470) mL/min/1.73 m^2^ (*p* < 0.0001) higher in marginal mean difference of eGFR, 0.542 (0.321, 0.764) mmol/L (*p* < 0.0001) higher in marginal mean difference of BUN, and 0.460 (0.166, 0.753) g/L (*p* = 0.002) in marginal mean difference of Alb than the non‐SMP group (Table 2 and Figure 4). Marginal mean difference of Hb was higher and marginal mean difference and TC were lower in the SMP group without statistical significance (*p* > 0.05).

## 4. Discussion

As Elendu et al. noted, CKD treatment aims to slow disease progression, manage complications, prevent ESKD, and improve quality of life. This requires a combination of lifestyle modifications, medications, and medical procedures, which is consistent with our SMP program, a comprehensive, proactive, and continually individualized approach [26].

In this retrospective cohort study, we discovered clinical differences between an SMP group and a non‐SMP group among patients with CKD stages 3‐4. Participating in SMP was a protective factor for mitigating the risk of CKD progression by as much as 30.9% and improvements in kidney function and nutrition‐related surrogate markers, benefiting CKD patients in stages 3‐4.

Clinical and epidemiologic studies have shown that healthy lifestyle and dietary patterns may have protective effects throughout the longitudinal progression of kidney‐related diseases, informing more effective management and treatment and supporting the effectiveness of our comprehensive SMP program in CKD populations [27, 28]. Even improvements in single nutrient intake could bring kidney benefits. For example, higher dietary potassium intake is associated with lower blood pressure, better kidney outcomes, and lower mortality [28]. Salt substitutes also improve blood pressure control and reduce all‐cause death and cardiovascular event risk in the general population compared with regular salt [28].

We believe our results are closely related to this SMP program. The distinct inflammatory uremic environment associated with CKD results in patients experiencing a decline in muscle protein synthesis and an acceleration of muscle breakdown. In CKD management, stringent nutritional and lifestyle interventions are regarded as vital measures to halt disease advancement. However, unscientific nutrition intervention methods frequently and unintentionally result in inadequate nutrient intake, coupled with intricate metabolic disorders in the human body, leading to nutritional disorders such as protein‐energy wasting (PEW) and cachexia, ultimately detrimental to renal outcomes [29–31]. Improper lifestyle adjustments, such as engaging in exercise methods and frequencies that exceed the body’s capacity, not only fail to enhance muscle mass but may also result in elevated urine protein levels and even increase the risk of fractures [32].

Our SMP program provides personalized recommendations tailored to each patient’s condition, aligned with professional guidelines and consensus. For instance, for patients with low muscle mass, we meticulously adjust their medication regimen based on various health indicators collected during each follow‐up visit. We also customize meal plans according to the patient’s preferences, incorporating the recommended intake of nutrients such as protein, energy, high‐quality protein (including animal‐ and plant‐based sources), and vitamin D as outlined in the guidelines. Furthermore, we encourage patients to engage in moderate resistance exercises, such as using elastic bands suited to their strength levels for about 30 min each day. Older patients can also incorporate simple exercises into their daily routines, such as repeatedly picking up bags of vegetables and fruits of appropriate weight while walking.

Through these measures, patients enrolled in our SMP program cultivate a strong sense of SMP, enabling them to actively monitor their condition and nutritional status and make necessary lifestyle changes, leading to improvements in their eGFR, BUN, and Alb levels. This easily adoptable plan may have effects on preventing adverse outcomes related to kidney function and nutrition. Our results also indicate that a more holistic SMP strategy may effectively mitigate the detrimental impacts of nutritional restrictions, yielding positive clinical results and striking a superior equilibrium between nutritional management, lifestyle modification, and kidney disease control. This methodology merits wider adoption. Nevertheless, to delve deeper into the mechanisms governing this equilibrium, future studies must incorporate a broader array of nutrition‐related indicators for comprehensive analysis.

Subgroup analysis demonstrated that there were no significant interaction effects between participation in SMP and subgroups defined by age, CKD stage, or diabetes status, but SMP had interactive effects within the hypertension subgroup. Participation in the SMP program was a protective factor among patients with/without diabetes and with/without hypertension. We believe that the effects of SMP on diabetes and hypertension were closely related to one of its distinct aspects: “risk factor management.” These results demonstrate the rationale and effectiveness of widespread application of SMP across a broad patient population.

Via a scope review of 10 years of real‐world data, this study offers evidence of the long‐term effects of CKD SMP. It suggests that engaging in a SMP program may reduce the risk of CKD progression by up to 30.9%. SMP is a special method that requires both the efforts of medical staff and high compliance from patients and their families. Additionally, attitudes and adherence from patients and their families exert considerable influence on SMP’s effects. CKD usually progresses in a persistent and chronic manner. Patients with CKD might not see obvious results in a short time, and therefore their enthusiasm may wane when they participate in SMP. Our study’s results indicate that long‐term SMP may improve CKD patients’ surrogate markers and prognosis through comprehensive and tailored SMP plans. These results improve medical staff’s confidence and stimulate both CKD patients’ attitudes and their behaviors.

## 5. Conclusion

Our SMP program mitigated the risk of CKD progression by 30.9% and potentially enhanced nutrition‐related surrogate markers, ultimately benefiting CKD patients in stages 3‐4.

## 6. Limitations

This study also has certain limitations: First, a retrospective cohort study could not cover data such as physical examinations, lifestyle habits, or medication adherence. The intergroup differences for these factors also could not be compared. Second, patients were grouped according to their records from the GPHCM outpatient clinic, as described in the Defining the Exposures section. Although we simplified the two groups to the best of our ability, we could not estimate SMP adherence. As such, there may have been measurement bias in our study. Third, the missing rates for some of the covariates were high and had strong associations with CKD prognosis, such as PCR. Fourth, although we collected a large amount of retrospective clinical patient data, it was still difficult to account for data from patients who had visited other healthcare settings and/or participated in other management programs. Fifth, this study was a single‐center retrospective study conducted in Southern China. Multicenter prospective studies with larger sample sizes are needed to extrapolate the results to a broader population. As a multidimensional treatment, SMP might change inflammation, immunoreactions, and other metabolism in the human body [33, 34]. Unfortunately, we could not collect biomarkers to verify the micromechanism of SMP. As such, more prospective studies are needed in the future.

## Author Contributions

Writing–original draft and formal analysis: Hui‐fen Chen; writing–review and editing: Xing‐le Liang; writing–review and editing: Yan Han; data curation: Yu‐han Shen; formal analysis: Xian‐long Zhang; data curation: Fang Tang; project administration: Li‐zhe Fu; funding acquisition and project administration: Yue‐yu Gu; project administration: Tao Zou; formal analysis: Xin‐dong Qin; methodology and supervision: Wen‐wei OuYang; conceptualization and funding acquisition: Xu‐sheng Liu; conceptualization and funding acquisition: Yi‐fan Wu. All authors provided critical revisions to the manuscript draft.

## Funding

This study was supported by the Incubation Program for the Science and Technology Development of Chinese Medicine Guangdong Laboratory (Project no. HQL2024PZ032), National Key Research and Development Program of China (Project no. 2019YFE0196300), the Fund of Guangdong Provincial Hospital of Chinese Medicine for Traditional Chinese Medicine Science and Technology Research (Project nos. YN2020ZWB05 and YN2023MS03), the Key Technologies Research and Development Program of Guangzhou Municipality of Agriculture and Social Development (no. 202206010102), and the Young Talent Program at the Guangdong Provincial Academy of Chinese Medical Sciences (no. SZ2022QN07).

## Disclosure

All authors approved the final manuscript for submission.

## Conflicts of Interest

The authors declare no conflicts of interest.

## Supporting Information

Supporting file 1 offered a comprehensive overview of the self‐management services and their distinct features at the Chronic Disease Management Outpatient Clinic of Guangdong Provincial Hospital of Chinese Medicine, which has methodically introduced its service model for chronic disease management. Supporting file 2 was overview of covariates. Supporting table 1 provided baseline characteristics of included patients. Supporting table 2 was the results of univariate Cox regression.

## Supporting information


**Supporting Information** Additional supporting information can be found online in the Supporting Information section.

## Data Availability

The data used to support the findings of this study are available on request from the corresponding author.
