# Supplementary material for: Self‐Management Improves Long‐Term CKD Prognosis: A 10‐Year Retrospective Cohort Study From China
Source: J Nurs Manag. 2026 Mar 3;2026:1228799. doi: 10.1155/jonm/1228799 (PMC12956840; doi:10.1155/jonm/1228799)
Supplement: Supplementary file 1 — Supporting Information Additional supporting information can be found online in the Supporting Information section. [file JONM-2026-1228799-s001.zip › Supplement table 1 baseline characteristics.docx]

**Supplementary Table 1: Baseline characteristics**

| Variables(n(%)/median(25th, 75th) | | Unmatched | | | | | After PSM | | | | |
| --- | --- | --- | --- | --- | --- | --- | --- | --- | --- | --- | --- |
|  |  | Overall (N=2,416) | Non-SMP (N=1,738) | SMP  (N=678) | *P* | SMD | Overall (N=1,160) | Non-SMP (N=580) | SMP  (N=580) | *P* | SMD |
| Age, years |  | 60.89 (49.91, 69.83) | 62.06 (50.66, 70.97) | 58.55 (47.51, 67.53) | <0.001 | 0.230 | 59.69 (48.64, 68.58) | 59.95 (48.42, 69.21) | 59.41 (48.66, 68.15) | 0.359 | 0.051 |
| Sex | Males | 1424 (58.94) | 1074 (61.80) | 350 (51.62) | <0.001 | 0.206 | 617 (53.19) | 305 (52.59) | 312 (53.79) | 0.724 | 0.024 |
|  | Females | 992 (41.06) | 664 (38.20) | 328 (48.38) |  |  | 543 (46.81) | 275 (47.41) | 268 (46.21) |  |  |
| eGFR, mL/min/1.73m^2^ | | 41.08 (28.33, 50.97) | 41.12 (29.31, 51.08) | 40.80 (26.37, 50.86) | 0.064 | 0.090 | 41.15 (27.66, 51.34) | 40.63 (27.64, 51.45) | 41.27 (27.70, 51.25) | 0.833 | 0.009 |
| Hb, g/L | | 122.00 (107.00, 136.00) | 122.00 (106.00, 136.00) | 121.00 (107.00, 136.00) | 0.614 | 0.048 | 124.00 (109.00, 138.00) | 123.00 (109.00, 138.00) | 124.00 (109.00, 137.00) | 0.697 | 0.017 |
| Alb, g/L | | 42.10 (37.80, 45.00) | 41.50 (37.20, 44.80) | 43.10 (39.30, 45.60) | <0.001 | 0.191 | 42.80 (38.80, 46.20) | 42.55 (37.70, 47.10) | 42.90 (39.50, 45.50) | 0.749 | 0.022 |
| UA, μmmol/L | | 452.00 (379.00, 532.00) | 458.00 (384.00, 542.75) | 436.00 (370.50, 508.00) | <0.001 | 0.222 | 439.50 (368.75, 509.00) | 440.00 (363.50, 509.00) | 438.50 (370.75, 509.25) | 0.982 | 0.017 |
| BUN, mmol/L | | 8.94 (7.02, 11.79) | 8.78 (7.00, 11.67) | 9.27 (7.08, 12.30) | 0.017 | 0.122 | 9.18 (7.07, 12.42) | 9.20 (7.21, 12.52) | 9.15 (7.03, 12.30) | 0.543 | 0.020 |
| TCO_2_, mmol/L | | 23.70 (21.70, 25.80) | 23.80 (21.80, 25.90) | 23.40 (21.30, 25.40) | 0.002 | 0.073 | 23.50 (21.40, 25.60) | 23.60 (21.37, 25.60) | 23.50 (21.58, 25.60) | 0.672 | 0.013 |
| LDL-C, mmol/L | | 3.14 (2.43, 3.98) | 3.11 (2.40, 3.94) | 3.23 (2.48, 4.08) | 0.184 | 0.022 | 3.23 (2.48, 4.16) | 3.23 (2.46, 4.16) | 3.24 (2.48, 4.16) | 0.990 | 0.010 |
| TC, mmol/L | | 4.96 (4.13, 5.85) | 4.93 (4.11, 5.86) | 5.00 (4.14, 5.84) | 0.572 | 0.214 | 5.03 (4.14, 5.94) | 5.02 (4.14, 5.96) | 5.05 (4.13, 5.89) | 0.998 | 0.003 |
| HDL-C, mmol/L | | 1.16 (0.94, 1.47) | 1.13 (0.91, 1.44) | 1.20 (1.00, 1.54) | <0.001 | 0.118 | 1.24 (1.00, 1.56) | 1.24 (1.00, 1.57) | 1.23 (1.00, 1.55) | 0.855 | 0.049 |
| CKD stage | CKD stage 3a | 972 (40.23) | 707 (40.68) | 265 (39.09) | 0.032 |  | 465 (40.09) | 236 (40.69) | 229 (39.48) | 0.705 |  |
|  | CKD stage 3b | 764 (31.62) | 567 (32.62) | 197 (29.06) |  |  | 345 (29.74) | 166 (28.62) | 179 (30.86) |  |  |
|  | CKD stage 4 | 680 (28.15) | 464 (26.70) | 216 (31.86) |  |  | 350 (30.17) | 178 (30.69) | 172 (29.66) |  |  |
| Etiology | Primary Glomerulonephritis | 564 (23.34) | 336 (19.33) | 228 (33.63) | <0.001 | 0.392 | 348 (30.00) | 171 (29.48) | 177 (30.52) | 0.825 | 0.072 |
|  | Hypertensive Renal Disease | 54 (2.24) | 31 (1.78) | 23 (3.39) |  |  | 34 (2.93) | 15 (2.59) | 19 (3.28) |  |  |
|  | Diabetic nephropathy | 112 (4.64) | 72 (4.14) | 40 (5.90) |  |  | 66 (5.69) | 34 (5.86) | 32 (5.52) |  |  |
|  | Other secondary kidney diseases | 178 (7.37) | 127 (7.31) | 51 (7.52) |  |  | 93 (8.02) | 51 (8.79) | 42 (7.24) |  |  |
|  | Unknown | 1508 (62.42) | 1172 (67.43) | 336 (49.56) |  |  | 619 (53.36) | 309 (53.28) | 310 (53.45) |  |  |
| Comorbidity | |  |  |  |  |  |  |  |  |  |  |
| With Hypertension | No | 783 (32.41) | 628 (36.13) | 155 (22.86) | <0.001 | 0.294 | 281 (24.22) | 140 (24.14) | 141 (24.31) | 1.000 | 0.004 |
|  | Yes | 1633 (67.59) | 1110 (63.87) | 523 (77.14) |  |  | 879 (75.78) | 440 (75.86) | 439 (75.69) |  |  |
| With Diabetes | No | 1494 (61.84) | 1154 (66.40) | 340 (50.15) | <0.001 | 0.334 | 631 (54.40) | 317 (54.66) | 314 (54.14) | 0.906 | 0.010 |
|  | Yes | 922 (38.16) | 584 (33.60) | 338 (49.85) |  |  | 529 (45.60) | 263 (45.34) | 266 (45.86) |  |  |
| With Hyperlipidemia | No | 1513 (62.62) | 1109 (63.81) | 404 (59.59) | 0.060 | 0.087 | 706 (60.86) | 359 (61.90) | 347 (59.83) | 0.508 | 0.042 |
|  | Yes | 903 (37.38) | 629 (36.19) | 274 (40.41) |  |  | 454 (39.14) | 221 (38.10) | 233 (40.17) |  |  |
| With Hyperuricemia | No | 1260 (52.15) | 939 (54.03) | 321 (47.35) | 0.004 | 0.134 | 581 (50.09) | 296 (51.03) | 285 (49.14) | 0.557 | 0.038 |
|  | Yes | 1156 (47.85) | 799 (45.97) | 357 (52.65) |  |  | 579 (49.91) | 284 (48.97) | 295 (50.86) |  |  |
| With Anemia | No | 1494 (61.84) | 1098 (63.18) | 396 (58.41) | 0.034 | 0.098 | 684 (58.97) | 336 (57.93) | 348 (60.00) | 0.511 | 0.042 |
|  | Yes | 922 (38.16) | 640 (36.82) | 282 (41.59) |  |  | 476 (41.03) | 244 (42.07) | 232 (40.00) |  |  |
| With CVDs | No | 1930 (79.88) | 1363 (78.42) | 567 (83.63) | 0.005 | 0.133 | 958 (82.59) | 480 (82.76) | 478 (82.41) | 0.938 | 0.009 |
|  | Yes | 486 (20.12) | 375 (21.58) | 111 (16.37) |  |  | 202 (17.41) | 100 (17.24) | 102 (17.59) |  |  |
| Medication in use | |  |  |  |  |  |  |  |  |  |  |
| ACEI/ARB | No | 1539 (63.70) | 1124 (64.67) | 415 (61.21) | 0.123 | 0.072 | 719 (61.98) | 354 (61.03) | 365 (62.93) | 0.545 | 0.039 |
|  | Yes | 877 (36.30) | 614 (35.33) | 263 (38.79) |  |  | 441 (38.02) | 226 (38.97) | 215 (37.07) |  |  |
| Calcium supplements | No | 2032 (84.11) | 1485 (85.44) | 547 (80.68) | 0.005 | 0.127 | 952 (82.07) | 478 (82.41) | 474 (81.72) | 0.818 | 0.018 |
|  | Yes | 384 (15.89) | 253 (14.56) | 131 (19.32) |  |  | 208 (17.93) | 102 (17.59) | 106 (18.28) |  |  |
| Sodium bicarbonate | No | 1716 (71.03) | 1336 (76.87) | 380 (56.05) | <0.001 | 0.452 | 729 (62.84) | 373 (64.31) | 356 (61.38) | 0.331 | 0.061 |
|  | Yes | 700 (28.97) | 402 (23.13) | 298 (43.95) |  |  | 431 (37.16) | 207 (35.69) | 224 (38.62) |  |  |
| Diuretics | No | 1983 (82.08) | 1391 (80.03) | 592 (87.32) | <0.001 | 0.198 | 1002 (86.38) | 501 (86.38) | 501 (86.38) | 1.000 | <0.001 |
|  | Yes | 433 (17.92) | 347 (19.97) | 86 (12.68) |  |  | 158 (13.62) | 79 (13.62) | 79 (13.62) |  |  |
| Chinese patent medicines | |  |  |  |  |  |  |  |  |  |  |
| Turbidity-removing | No | 1648 (68.21) | 1089 (62.66) | 559 (82.45) | <0.001 | 0.455 | 917 (79.05) | 453 (78.10) | 464 (80.00) | 0.471 | 0.047 |
|  | Yes | 768 (31.79) | 649 (37.34) | 119 (17.55) |  |  | 243 (20.95) | 127 (21.90) | 116 (20.00) |  |  |
| Tonifying | No | 2024 (83.77) | 1481 (85.21) | 543 (80.09) | 0.003 | 0.136 | 951 (81.98) | 475 (81.90) | 476 (82.07) | 1.000 | 0.004 |
|  | Yes | 392 (16.23) | 257 (14.79) | 135 (19.91) |  |  | 209 (18.02) | 105 (18.10) | 104 (17.93) |  |  |

Notes: Estimated glomerular filtration rate: eGFR; Hemoglobin: Hb; Albumin: ALB; uric acid: UA; blood urea nitrogen:BUN; total carbon dioxide: TCO2; low-density lipoprotein cholesterol: LDL-C; total cholesterol: TC; high-density lipoprotein cholesterol:HDL-C. Primary glomerulonephritides included chronic nephritis, nephropathy syndrome and IgA nephropathy. Other secondary nephrosis included systemic lupus erythematosus nephritis, Henoch-Schonlein purpura, hepatitis B virus-associated nephritis, obstructive nephropathy, etc. CVDs, cardiovascular diseases;Angiotensin converting enzyme inhibitors: ACEI; angiotensin receptor blocker: ARB.
