# Supplementary material for: Self‐Management Improves Long‐Term CKD Prognosis: A 10‐Year Retrospective Cohort Study From China
Source: J Nurs Manag. 2026 Mar 3;2026:1228799. doi: 10.1155/jonm/1228799 (PMC12956840; doi:10.1155/jonm/1228799)
Supplement: Supplementary file 1 — Supporting Information Additional supporting information can be found online in the Supporting Information section. [file JONM-2026-1228799-s001.zip › Supplement table 2 Univariate Cox regression.docx]

**Supplementary Table 2 Univariate Cox regression**

| Variables |  | HR (95%CI) | *P* | *P for PH test* |
| --- | --- | --- | --- | --- |
| **Group** | Non-SMP | Ref |  |  |
|  | SMP | 0.716(0.571,0.897) | 0.004 | 0.085 |
| **Age, years** |  | 0.987(0.979,0.995) | 0.001 | 0.943 |
| **Sex** | Males | Ref |  |  |
|  | Females | 0.719(0.573,0.903) | 0.005 | 0.182 |
| **eGFR, mL/min/1.73m^2^** |  | 0.933(0.925,0.942) | <0.001 | 0.179 |
| **Hb, g/L** |  | 0.976(0.971,0.981) | <0.001 | 0.567 |
| **Alb, g/L** |  | 0.949(0.935,0.962) | <0.001 | 0.067 |
| **UA, μmmol/L** |  | 1.001(1.000,1.002) | 0.021 | 0.794 |
| **BUN, mmol/L** |  | 1.106(1.092,1.121) | <0.001 | 0.001 |
| **TCO_2_, mmol/L** |  | 0.909(0.876,0.943) | <0.001 | 0.107 |
| **LDL-C, mmol/L** |  | 1.118(1.056,1.185) | <0.001 | 0.406 |
| **TC, mmol/L** |  | 1.134(1.078,1.193) | <0.001 | 0.690 |
| HDL-C, mmol/L |  | 0.931(0.729,1.189) | 0.566 | 0.020 |
| **CKD stage** | CKD stage 3a | Ref |  |  |
|  | CKD stage 3b | 2.346(1.648,3.340) | <0.001 | 0.632 |
|  | CKD stage 4 | 7.430(5.433,10.160) | <0.001 |  |
| **Etiology** | Primary Glomerulonephritis | Ref |  |  |
|  | Hypertensive Renal Disease | 1.088(0.594,1.992) | 0.784 | 0.092 |
|  | Diabetic nephropathy | 2.948(1.964,4.426) | <0.001 |  |
|  | Other secondary kidney diseases | 1.012(0.634,1.616) | 0.960 |  |
|  | Unknown | 0.990(0.761,1.288) | 0.942 |  |
| Comorbidity |  |  |  |  |
| **With Hypertension** | No | Ref |  | 0.106 |
|  | Yes | 2.001(1.467,2.730) | <0.001 |  |
| **With Diabetes** | No | Ref |  | 0.062 |
|  | Yes | 1.923(1.532,2.414) | <0.001 |  |
| With Hyperlipidemia | No | Ref |  | 0.022 |
|  | Yes | 1.212(0.967,1.520) | 0.095 |  |
| With Hyperuricemia | No | Ref |  | 0.921 |
|  | Yes | 1.076(0.860,1.346) | 0.522 |  |
| **With Anemia** | No | Ref |  | 0.108 |
|  | Yes | 2.241(1.788,2.809) | <0.001 |  |
| **With CVDs** | No | Ref |  | 0.419 |
|  | Yes | 1.583(1.210,2.070) | 0.001 |  |
| Medication in use |  |  |  |  |
| ACEI/ARB | No | Ref |  | 0.874 |
|  | Yes | 1.224(0.977,1.533) | 0.079 |  |
| Calcium supplements | No | Ref |  | 0.960 |
|  | Yes | 0.962(0.717,1.291) | 0.796 |  |
| **Sodium bicarbonate** | No | Ref |  | 0.903 |
|  | Yes | 1.277(1.019,1.601) | 0.034 |  |
| **Diuretics** | No | Ref |  | 0.528 |
|  | Yes | 2.335(1.782,3.060) | <0.001 |  |
| Chinese patent medicines |  |  |  |  |
| **Turbidity-removing** | No | Ref |  | 0.183 |
|  | Yes | 1.762(1.377,2.253) | <0.001 |  |
| Tonifying | No | Ref |  | 0.540 |
|  | Yes | 1.168(0.889,1.534) | 0.265 |  |

Note: Estimated glomerular filtration rate: eGFR; hemoglobin: Hb; albumin: Alb; uric acid: UA; blood urea nitrogen:BUN; total carbon dioxide: TCO2; low-density lipoprotein cholesterol: LDL-C; total cholesterol: TC; high-density lipoprotein cholesterol: HDL-C. Primary glomerulonephritides included chronic nephritis, nephropathy syndrome and IgA nephropathy. Other secondary nephrosis included systemic lupus erythematosus nephritis, Henoch-Schonlein purpura, hepatitis B virus-associated nephritis, obstructive nephropathy, etc. Angiotensin converting enzyme inhibitors: ACEI; angiotensin receptor blocker: ARB.
