# Supplementary material for: Self‐Management Improves Long‐Term CKD Prognosis: A 10‐Year Retrospective Cohort Study From China
Source: J Nurs Manag. 2026 Mar 3;2026:1228799. doi: 10.1155/jonm/1228799 (PMC12956840; doi:10.1155/jonm/1228799)
Supplement: Supplementary file 1 — Supporting Information Additional supporting information can be found online in the Supporting Information section. [file JONM-2026-1228799-s001.zip › Supplementary file 1.docx]

**Supplementary file 1**

The Introduction of Self-management Services at Guangdong Provincial Hospital of Chinese Medicine

**Features of self-management services**

Our Chronic Disease Management Clinic offers a special model for a nurse-led outpatient clinic that includes several characteristics: Firstly, the outpatient clinic does not solely utilize pharmacological interventions as a means of treatment; rather, in order to thwart CKD progression, it emphasizes various forms of education and training which are provided to patients with chronic kidney disease (CKD) by healthcare professionals (including physicians and nurses) to modify lifestyles and prevent and control potential risk factors. Secondly, our clinic emphasizes patients' enthusiasm and initiative in the treatment process. Specialist nurses will educate CKD patients on self-management via various forms of instruction including bulletin boards, health lectures, and online communication through scientific information sharing tools (i.e., our official WeChat^[[1]](#footnote-0)^ account, WeChat channels and TikTok). Thirdly, our clinic proposes a working regime focused on nurse accountability. Specialist nurses are responsible for building health records for CKD patients upon their first visit. Specialist nurses record long-term data and provide health education for CKD patients via modern communication tools (phone contact, SMS messaging and WeChat) after the first consultation. This ensures that patients are able to receive effective care and guidance following outpatient consultation. The persistent treatment reduces the likelihood of loss of follow-up, and facilitates accurate health record-keeping.

Self-management is the core operational concept in the Chronic Disease Management Clinic. It transforms the traditional paternalistic model into a more equitable and collaborative model between patients, their caregivers and healthcare providers to collaborate and co-manage the chronic disease during diagnostic and therapeutic procedures. CKD self-management involves several components, including disease therapy, behavior management, role management and emotion management.

**Theoretical Framework for Nursing Care in Chronic Disease Management Clinics**

The "5A Model" is a theoretical framework guiding nursing care in the Chronic Disease Management Clinic. It is a standardized model of care consisting of five steps: assessment, advising, agreement, assistance and arranging.

The first step is *assessment*. Specialist nurses conduct a comprehensive inquiry. This consists of a series of questionnaires and examination devices to investigate and assess patients’ disease condition (etiology, comorbidity, and family history), such as medication used, daily routine, dietary habits and nutritional status, lifestyle, disease awareness, and psychological status. This allows for a detailed and comprehensive understanding of the disease condition and the discovery of any potential risks in need of intervention.

The second step is *advising*. Utilizing the assessment results, specialist nurses combine the latest domestic and international medical guidelines and consensus, as well as the patient's individual circumstances, to provide the patient with relevant advice on lifestyle adjustments, dietary guidance, psychological support, and management of the disease and medication.

The third step is *agreement*. Specialist nurses determine the management objectives and specific methods of implementation with patients and their caregivers based on the recommendations from the second step. Additionally, the specialist nurses develop a self-management training program.

The fourth step is *assistance*. Based on the advice provided in the second step, specialist nurses conduct face-to-face interviews, group lectures, and scientific information sharing with each patient and their caregivers, in order to achieve specific management goals. For example, we disseminate relevant information and training courses (online and offline) to patients.

The final step is *arranging*. This is comprised of two primary components. Firstly, specialist nurses act as intermediaries to transfer patients to other specialists such as psychologists or nutritionists, if necessary. Secondly, specialist nurses are responsible for arranging long-term follow-up schedules to ensure effective self-management and desirable treatment efficacy.

**Self-management plan development**

In the Chronic Disease Management Clinic, nephrologists serve the important role of making clinical diagnoses and determining treatment plans. Then, the patient is referred to a specialist nurse, who is responsible for formulating a self-management plan according to each CKD patient’s specific conditions.

A self-management plan must consist of three distinct components:

1. Five elements: regular visits, medication adjustments, nutritional intervention, lifestyle

modification, education/behavior guidance

1. Regular visits

A nephrologist will determine the frequency of follow-up visits at 3-6 month intervals according to the patient's condition. The specialist nurses will make appointments for each of the patient's visits, and provide them with self-management services according to the doctor's medical advice.

1. Medication adjustment

An attending physician will review and adjust medications in use based on the patient's condition. Then, a specialist nurse will explain the treatment plan to the patient, ensuring that they are cognizant of the pharmacological effects, dosage, and administration of each medication. Additionally, they will periodically assess the patient's medication adherence under the direction of the physician.

1. Nutritional intervention

It is the responsibility of the specialist nurses and nutritionists to deliver nutrition training courses to patients, including instructions on the intake of nutrients that have been identified as being of major concern, an overview of their nutritional status and required nutrients, and whether commonly consumed foods are suitable for them. Then, specialist nurses and nutritionists will develop an initial personalized nutrition plan in collaboration with patients and their caregivers. Next, the specialist nurses will be responsible for regularly assessing nutrition and monitoring diet intake, and updating the personalized nutrition plans at each visit. For instance, patients are required to implement an initial nutrition plan for a period of one month, and to record their daily dietary intake using either a three-day dietary record (at home, comprised of a weekend and two workdays) or a 24-hour dietary review (provided by a specialist nurse at each visit). Then, the specialist nurses will analyze the dietary records to evaluate the appropriateness of food types, nutrient intake volume, and time of intake. Additionally, the specialist nurses will assess nutrition status via a series of questionnaires and anthropometrics. In combination with the results above and patients’ regular diets, we will provide dietary suggestions for them to improve their diets.

1. Lifestyle modification

Specialist nurses will conduct a body composition analysis for the patient to asses muscle mass and fat mass. Based on the results, experts on lifestyle will provide exercise advice and assist the patient in selecting an appropriate exercise mode and intensity. The nurses will monitor and assess the patient's progress regularly. Additionally, the nurses will provide personalized instructions on daily routine according to the patient's lifestyle habits.

1. Education and behavior guide

The objective of the education and behavior guide is to provide patients with comprehensive information about CKD through various media, including face-to-face interviews, a WeChat official account, WeChat channels and TikTok. The aim is to promote patients' understanding of their disease and the associated self-management skills, thereby improving their self-management capacity. Additionally, specialist nurses will engage in regular communication with patients to address any negative emotions, such as anxiety or depression. In cases where patients exhibit clear psychological distress, an external psychology department will be consulted for further assistance.

1. Risk factor management (blood pressure, blood glucose, lipids and uric acid)

As CKD progresses, patients may develop complications, including hypertension, hyperglycemia, hyperlipidemia, and hyperuricemia. If these complications are not properly managed, they can accelerate the progression of CKD. To promote patients' self-management capacity and to better control high-risk factors, the specialist nurses will provide patients with comprehensive information on the administration of pharmaceuticals for the treatment of various complications, in accordance with the directives of their physicians. At the same time, the specialist nurses will teach them how to self-monitor their blood pressure and blood glucose.

1. Symptom management

Symptoms associated with CKD include edema, insomnia, constipation, and gout attacks. Specialist nurses will provide appropriate self-management training courses to CKD patients according to their specific conditions. For instance, nurses will instruct patients on controlling their daily water intake to manage edema. For patients with insomnia, a nurse will provide training on various techniques to facilitate sleep, and assist them in potential triggers in their daily lives. For patients with constipation, a nurse will educate them on dietary and bowel habit modifications to improve their symptoms. For patients with frequent gout attacks, a nurse will instruct them on the appropriate use of medication for hyperuricemia, and the avoidance of purine-rich foods and habits.

**Daily workflow for CKD self-management services**

The daily workflow for the CKD department at the Chronic Disease Management Clinic consists of two sessions: the initial consultation and the follow-up consultation.

1. Initial consultation session

In this session, the attending physicians formulate a treatment plan based on the patient's condition, and set initial focal points for self-management intervention. Then, the patient is transferred to a specialist nurse to sign an informed consent form. The specialist nurse collects their fundamental information such as demographic information, etiology and comorbidity, disease awareness and lifestyle, via a face-to-face interview and questionnaire. Then, the specialist nurse provides one-on-one self-management training based on their individual characteristics. During the training, the specialist nurse explains the disease's pathogenesis, potential treatment plans, self-management skills, and other key information. To ensure effective communication between the patient and the healthcare team, specialist nurses will also establish remote communication pathways to provide continuous health guidance and support.

1. Follow-up consultation session

We have implemented a follow-up appointment system to ensure an organized and efficient treatment process. The attending physicians adjust the treatment plan in accordance with the patient's condition, and determine the follow-up appointment frequency. This provides a comprehensive medication and follow-up schedule for the nurses’ reference. Then, the specialist nurses are responsible for scheduling telephone appointments with the CKD patients, and ensuring that they are able to attend the appointments on time. The nurses also inform the patient of the training and assessment tasks that need to be completed during the follow-up appointment, and supervise these tasks to the greatest extent possible. The follow-up process can be employed to boost the patient's capacity for self-management, and to guarantee the efficacy of the self-management.

The follow-up consultation is a long-term and ongoing process. The specialist nurses continue tracking the CKD patients who participate in the self-management program until the occurrence of either end-stage kidney disease (ESRD) or death, the implementation of renal replacement therapy (dialysis or kidney transplantation), or loss of follow-up.

If a patient withdraws from the self-management program, the specialist nurses will investigate the reasons for the withdrawal in order to adjust and optimize future follow-up work.

1. WeChat is a popular app in China with many functions, including messaging, social media, and content distribution. [↑](#footnote-ref-0)
