# Supplementary material for: Self‐Management Improves Long‐Term CKD Prognosis: A 10‐Year Retrospective Cohort Study From China
Source: J Nurs Manag. 2026 Mar 3;2026:1228799. doi: 10.1155/jonm/1228799 (PMC12956840; doi:10.1155/jonm/1228799)
Supplement: Supplementary file 1 — Supporting Information Additional supporting information can be found online in the Supporting Information section. [file JONM-2026-1228799-s001.zip › Supplementary file 2.docx]

**Supplementary file 2 Covariate Overview**

Demographic characteristics (patient ID, age, and sex (male/female)), clinical diagnosis information (CKD stage, etiology and comorbidities), and laboratory measurements. Etiology was classified as primary glomerulopathy, hypertensive nephropathy, diabetic nephropathy, other secondary kidney diseases (including infectious or autoimmune kidney diseases, obstructive nephropathy, uric acid nephropathy, and pyelonephritis), and unknown etiology. Comorbidities including hypertension, diabetes, hyperlipidemia, hyperuricemia, anemia, and cardiovascular diseases (CVDs) were identified based on diagnostic records, medication records, and patient self-report. Medication in use included ACEI/ARB, calcium supplements, sodium bicarbonate, diuretics and Chinese patent medicine (were widely used in China, including Chinese patent medicines with turbidity-removing and tonifying efficacy mainly). Laboratory covariates consisted of serum creatinine (SCr), eGFR, hemoglobin (Hb), serum albumin (Alb), blood urea nitrogen (BUN), total carbon dioxide (TCO_2_), Uric Acid (UA), phosphorous (P), calcium (Ca^2+^), potassium (K^+^), sodium (Na^+^), low-density lipoprotein cholesterol (LDL-C), total cholesterol (TC), high-density lipoprotein cholesterol (HDL-C), fasting blood glucose (FBG), and urine protein/creatinine ratio (PCR).
